# Molecular Identification of *Sarcocystis rileyi* and *Sarcocystis* sp. (Closely Related to *Sarcocystis wenzeli*) in Intestines of Mustelids from Lithuania

**DOI:** 10.3390/ani13030467

**Published:** 2023-01-29

**Authors:** Petras Prakas, Darija Moskaliova, Donatas Šneideris, Evelina Juozaitytė-Ngugu, Evelina Maziliauskaitė, Saulius Švažas, Dalius Butkauskas

**Affiliations:** Nature Research Centre, Akademijos Str. 2, LT-08412 Vilnius, Lithuania

**Keywords:** *Sarcocystis rileyi*, mustelidae, anseriformes, chickens, definitive host, *ITS1*, molecular identification

## Abstract

**Simple Summary:**

Protozoan parasites of the genus *Sarcocystis* are characterised by a two-host prey–predator life cycle. To date, more than 25 *Sarcocystis* species have been confirmed to form sarcocysts in muscles and CNS of birds. Avian *Sarcocystis* species are transmitted via predatory birds, placental mammals, and opossums. The objective of the study was to examine the role of predatory mammals of the family Mustelidae in the transmission of avian *Sarcocystis* spp. by means of molecular methods. In total, 115 small intestine samples of mustelids collected in Lithuania were tested for the presence of *Sarcocystis* spp. using anseriforms and domestic fowl (*Gallus domesticus*) as their intermediate hosts. Based on the DNA sequence analysis, *S*. *rileyi* known as forming macrocysts in muscles of ducks was detected in 11.3% of examined small intestine samples and *Sarcocystis* sp. was identified in two samples. The latter species was most closely related to *Sarcocystis* spp. isolates infecting chickens and causing encephalitis. This is the first report of avian *Sarcocystis* identified by molecular methods in the small intestines of mustelids, indicating the significance of these small predators for the spreading of *Sarcocystis* spp. using birds as intermediate hosts. Based on current knowledge, canids and mustelids are most likely the definitive hosts of *S*. *rileyi* in Europe.

**Abstract:**

The genus *Sarcocystis* is a group of numerous protozoan parasites having a two-host life cycle. Based on laboratory experiments and/or phylogenetic analysis results it was shown that seven *Sarcocystis* spp. producing sarcocsyts in bird tissues are transmitted via predatory placental mammals. To date the role of small mammals of the family Mustelidae in the distribution of avian *Sarcocystis* spp. have not been studied. During the current investigation, intestinal mucosa scrapings of 115 mustelids belonging to five species were tested for *S. albifronsi, S. anasi*, *S. rileyi*, and *S. wenzeli* infecting anseriforms and chickens. Microscopically, free sporocysts, sporulating oocysts, and loose oocysts were found in 61 samples (53.0%). Using nested PCR targeting the *ITS1* region and sequencing, *S*. *rileyi* was confirmed in eight American minks, two European polecats and single European badger. *Sarcocystis* sp. was identified in one American mink and one European pine marten. Based on the partial *ITS1* region this parasite showed that 100% identity to pathogenic *Sarcocystis* sp. caused a fatal infection in backyard chickens from Brazil. Phylogenetically, the *Sarcocystis* sp. identified in our study was most closely related to *S*. *wenzeli* parasitising domestic fowl (*Gallus domesticus*).

## 1. Introduction

Members of the genus *Sarcocystis* (Apicomplexa: Sarcocystidae) are protozoan parasites distributed worldwide. The genus *Sarcocystis* has a broad host spectrum encompassing mammals, birds and reptiles. These parasites are distinguished by an obligatory prey–predator two-host life cycle [1]. Sarcocysts are found mainly in muscles or CNS of intermediate hosts, while endogenous sporulation of oocysts take place in the intestine of the definitive host [2]. To date more than 200 *Sarcocystis* species are known, some of them being pathogenic for their intermediate host [1,3]. *Sarcocystis* species are morphologically characterised and described in intermediate hosts, while oocyst and sporocyst of parasite species found in definitive hosts can be differentiated only by molecular methods [4].

Birds serve as intermediate hosts for more than 25 known species of *Sarcocystis* [5]. Based on laboratory experiments, predatory birds, placental mammals, and opossums of the genus *Didelphis* are definitive hosts of *Sarcocystis* species forming sarcocysts in tissues of birds [1,6]. Phylogenetic results indicate that seven species, *S. albifronsi*, *S. anasi*, *S. atraii*, *S. chloropusae*, *S. cristata*, *S. rileyi*, and *S. wenzeli* are transmitted via predatory mammals of the order Carnivora [1,5,7,8,9,10,11]. Three species, *S. albifronsi, S. anasi*, and *S. rileyi* infect muscles of ducks and geese [7,8,12,13], *S. wenzeli* are found in chickens [11], *S. atraii* and *S. chloropusae* were described in birds of order Gruiformes [9,10], and *S. cristata* was detected in the representative of order Musophagiformes [5]. Of these seven species, three, *S. albifronsi*, *S. anasi*, and *S. rileyi*, were confirmed in Lithuania [7,12].

*Sarcocystis* species are mostly genetically characterised at nuclear *18S* rDNA, *28S* rDNA, *ITS1*, and mitochondrial *cox1* [1,5]. The choice of genetic loci for the identification of *Sarcocystis* species depends on their hosts [14]. For instance, *cox1* is most appropriate for the differentiation of *Sarcocystis* species employing ruminants as intermediate hosts [15,16]. Avian *Sarcocystis* spp. could be differentiated on the basis of the *28S* rDNA and *ITS1*, and the *ITS1* is more variable of these two genetic markers [1,17]. By contrast, *18S* rDNA and *cox1* appeared to be insufficiently variable for the discrimination of some *Sarcocystis* spp. employing birds as intermediate hosts [17]. 

Representatives of the family Mustelidae are widespread in Lithuania [18,19]. They occur in all habitats and with nine species compose most diverse family of the order Carnivora [19,20,21,22,23]. It has been shown that mustelids play a significant role in transmitting *Sarcocystis* species forming sarcocysts in rodents and ungulates [16,24]. 

So far, no research has been carried out to find out whether mustelids can contribute to the transmission of *Sarcocystis* species those intermediate hosts are birds. The invasive American mink (*Neovison vison*) is an important predator of ducks in wetland habitats of Finland and Denmark [25,26]. In Lithuania and Latvia, the mass killing of incubating females of mallard (*Anas platyrhynchos*), common pochard (*Aythya farina*), and tufted duck (*Aythya fuligula*) by American mink was recorded, particularly on small islands of lakes where ducks breed almost colonially [27]. Birds are an important food component also for other mustelid species in Lithuania [28]. The diet of mustelids includes eggs, young and adult individuals of various waterbird species, and also birds found dead, particularly in winter [18,19]. Taking into account the diet of mustelids and their abundance in Lithuania, the aim of this study was to investigate the potential role of mustelids in spreading *Sarcocystis* species using birds as intermediate hosts. To achieve this objective, intestinal mucosa scrapings of mustelids collected in Lithuania were tested for the presence of *S. albifronsi*, *S. anasi*, *S. rileyi*, and *S. wenzeli* by means of molecular methods. 

## 2. Materials and Methods

### 2.1. Sample Collection and Isolation of Oocysts/Sporocysts 

A total of 115 animals (61 American mink, 26 European pine marten *Martes martes*, 18 European polecat *Mustela putorius*, 6 European badger *Meles meles*, and 4 Beech marten *Martes foina*) from the Mustelidae family were collected in accordance with national and institutional guidelines from licensed third parties. The animals were legally hunted mainly in southern, eastern, and central Lithuania between 2017 and 2021, in September–April and were kept frozen at –20 °C. The small intestine was removed from the animals and cut lengthwise. The intestinal epithelium was lightly scraped using a scalpel and suspended in 50 mL of water. The isolation of oocysts/sporocysts of *Sarcocystis* spp. was performed using previously described methodology [24].

### 2.2. Molecular Identification and Phylogenetic Analysis

Genomic DNA extraction was performed using a GeneJET Genomic DNA Purification Kit (Thermo Fisher Scientific Baltics, Vilnius, Lithuania) according to the manufacturer’s instructions. The DNA samples were kept frozen at −20 °C until further molecular analysis. 

Nested PCR amplification of internal transcribed spacer 1 (*ITS1*) partial sequences was performed. In the first step, forward SU1F (5’- GATTGAGTGTTCCGGTGAATTATT -3’) and reverse 5.8SR2 (5’- AAGGTGCCATTTGCGTTCAGAA -3’) primer pair was used [29]. Whereas in the second step, two primer pairs, GsSrilF2 (5’- ACGTTGTTCTATATTATGTGACCATT -3’)/GsSrilR2 (5’- TACTATAGAGGTGAAAGGGAGGTGA -3’) and AZVF1 (5’- TCAAAACGTCCAAATAATGGTAT -3’)/AZVR1 (5’- ACACATTCCTACTGCCTTCCAC -3’) were used. The following primers were designed using the Primers 3 Plus program [30]. In silico, the first primer pair was chosen to amplify *ITS1* fragments of *S*. *rileyi*, while the second primer pair was selected to amplify fragments of *S. albifronsi*, *S. anasi*, and *S. wenzeli*. Positive controls (DNA of *S. albifronsi*, *S. anasi* and *S. rileyi* extracted from single sarcocysts) were used in each set of PCRs. Three negative controls (nuclease free water instead of target DNA) were used: one for the first amplification step and two for the second step of nested PCR. The third negative control was obtained by transferring two µL from the negative control of the first amplification step to the negative control of the second amplification step.

PCR reactions were carried out using DreamTaq PCR Master Mix (Thermo Fisher Scientific Baltics, Vilnius, Lithuania) according to the manufacturer’s instructions. The PCR cycling conditions were as followed: initial denaturation for 5 min at 95 °C, 35 cycles of 45 s at 94 °C, 45 s at 55, 57, or 63 °C depending on the primer pair, 60 s at 72 °C, and final extension for 10 min at 72 °C. PCR products were observed in agarose gel and purified using Exonuclease I and FastAP Thermosensitive Alkaline Phosphatase (Thermo Fisher Scientific Baltics, Vilnius, Lithuania). Amplified products of the second nested PCR step were sequenced directly with the 3500 Genetic Analyzer (Applied Biosystems, Foster City, CA, USA) using the same forward and reverse primers as for PCR. The obtained *ITS1* sequences were deposited in GenBank with accession numbers OP970969–OP970981. 

The examined sequences were combined into single fragments, truncated excluding primer-binding nucleotide positions, checked manually for ambiguously placed nucleotides, and compared by BLAST (http://blast.ncbi.nlm.nih.gov/, accessed on 30 November 2022). For phylogenetic analysis sequences were aligned using MUSCLE algorithm loaded in MEGA7 software [31]. Selection of the evolutionary model best fit to the obtained multiple-sequence alignment and construction of phylogenetic tree by Bayesian methods were conducted with the help of TOPALi v2.5 software [32].

### 2.3. Data Analysis

Sterne’s exact method [33] was used to compute 95% confidence interval (CI) for the prevalence of *Sarcocystis* spp. in host species and in animals hunted during different months. Differences in the detection of *Sarcocystis* species in the examined mustelid species were evaluated using a Chi-squared test. The unconditional exact test was used to compare *S*. *rileyi* prevalence in animals collected in different months [34]. Statistical tests were carried out using the Quantitative Parasitology 3.0 software [35]. 

## 3. Results

### 3.1. Microscopical Examination of Sarcocystis spp. Oocysts/Sporocysts

**Figure 1:**
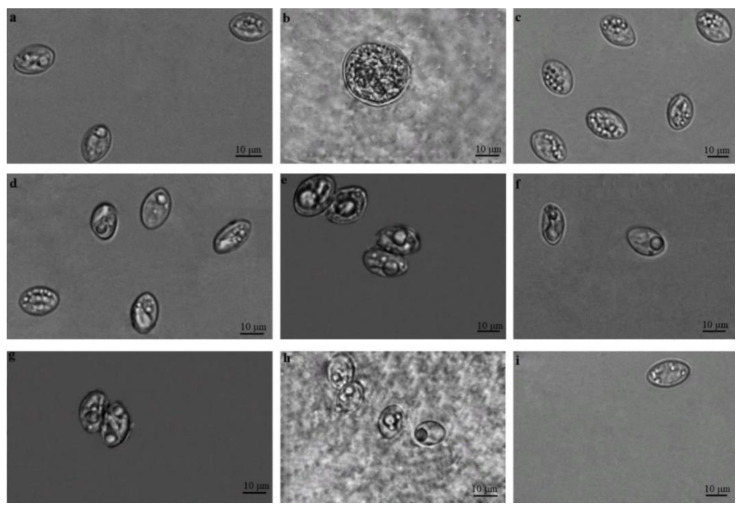
Oocysts/sporocysts found in small intestine mucosal scrapings of Mustelidae species. (**a**,**c**,**d**,**f**,**h**,**i**) Sporocysts. (**b)** Oocysts. (**e**,**g**,**h**) Sporulated oocysts. *Sarcocystis* spp. from American mink (**a**,**b**), European pine marten (**c**), European polecat (**d,e**), European badger (**f**,**g**), and Beech marten (**h**,**i**).

*Sarcocystis* spp. sporocysts and/or oocysts were noticed in the intestinal epithelium of the small intestines of all five species of mustelids analysed in the current study (Figure 1). In some samples, only a few (one-five) sporocysts and/or oocysts were detected in the area of the 24 × 24 mm coverslip, while in other samples, numerous parasites of different stages were found and it was even difficult to count the exact number of oocysts/sporocysts. 

Under a light microscope, oocysts and/or sporocysts of *Sarcocystis* spp. were detected in 61 of 115 (53.0%, 95% CI = 43.9%–62.2%) analysed samples (Table 1). The differences in *Sarcocystis* spp. detection rates among five predator species were insignificant (χ2 = 4.47, df = 4, *p* = 0.349). It should be noted that free sporocysts were seen more often than sporulating oocysts or loose oocysts. Free sporocysts of *Sarcocystis* spp. measured 11.8 × 8.3 μm (7.1–14.5 × 6.5–10.9 μm; *n* = 450), whereas ellipsoidal sporulated oocysts were thin-walled, contained two sporocysts, and measured 18.1 × 15.6 μm (17.5–19.0 × 15.1–15.9; *n* = 130). Oocysts measured 19.9 × 17.1 μm (13.9–23.5 × 12.0–22.4; *n* = 46) and were seen in the intestinal mucosa of American mink, European pine marten, and European polecat. The morphometric sizes of sporocysts and oocysts found in different predator species overlapped (Table 1). Further molecular analysis was used for the identification of selected parasite species in the examined specimens of intestine mucosal scrapings. 

### 3.2. Sarcocystis Species Identification and Their Distribution in Intestine Samples of Mustelids

Based on the nested PCR targeting the partial *ITS1* region, the sequencing of amplified products, and the comparison of obtained sequences, two *Sarcocystis* species were identified (Table 2). The more common *S*. *rileyi* was confirmed in 11 samples (9.6%, 95% CI = 5.1%–16.4%). This *Sarcocystis* species forming macrocysts in ducks [12,13] was identified in eight American minks (13.1%, 95% CI = 6.2%–24.4%), two European polecats (11.1%, 95% CI = 20.0%–33.0%), and a single European badger (16.7%, 95% CI = 8.6%–58.9%). Meanwhile, undescribed *Sarcocystis* sp. was established in one American mink (1.6%, 95% CI = 0.9%–8.7%) and one European pine marten (3.8%, 95% CI = 0.2%–18.8%). The *ITS1* fragments of *S*. *rileyi* and *Sarcocystis* sp. were amplified using GsSrilF2/GsSrilR2 and AZVF1/AZVR1 primer pairs, respectively. Notably, *S*. *rileyi* and *Sarcocystis* sp. were identified in different animals and the overall prevalence of *Sarcocystis* spp. in the intestinal mucosa of mustelids accounted for 11.3% (95% CI = 6.3%–18.6%). 

Eleven 611–612 bp-long *ITS1* sequences of *S*. *rileyi* determined in the present investigation displayed 99.18%–100% identity between each other. Of the 11 sequences, 9 identical ones (OP970971-79) showed an 100% match with other sequences of *S*. *rileyi* available in GenBank (GU188427, HM185744, KJ396584, MZ151434, MZ468639-40, and LT992314-16). The remaining two sequences of *S*. *rileyi* obtained in the current work (OP970980-81) differed from the most common haplotype by two nucleotide substitutions and one deletion and by two nucleotide substitutions, respectively. The sequences of *S*. *rileyi* obtained from mucosal scrapings of mustelids showed 91.22%–92.34% similarity to *S*. *atraii* from the common coot (*Fulica atra*) from Egypt and less than 74% similarity compared with the sequences of other *Sarcocystis* species.

Two 817 bp-long *ITS1* sequences of *Sarcocystis* sp. LT-2022 obtained in the present work (OP970969-70) did not differ from each other. These two *ITS1* sequences showed 100% identity with the sequence of *Sarcocsytis* sp. Chicken-2016-DF-BR (MN846302) isolated from brain tissues of two chickens in Brazil [36], 98.17%–98.66% similarity with the sequences of *S*. *wenzeli* (MT756994-98) parasitising chickens [11], and 95.49%–96.99% similarity with the sequences (OP490606-9, OP490613-4) obtained from pooled samples of the brain, pectoral muscle, lung, and heart of native village chickens in Malaysia. 

**Figure 2:**
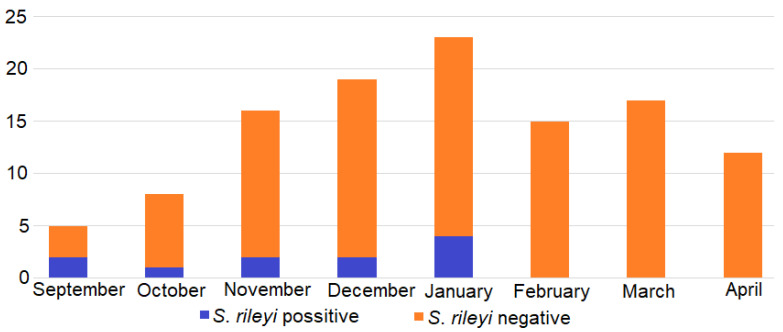
The molecular identification of *S*. *rileyi* in the mucosal scrapings of the mustelids collected during different months in Lithuania.

We observed seasonal changes in the abundance of *S*. *rileyi* in our sample (Figure 2). *S. rileyi* was determined by molecular methods in one animal hunted in October, in two animals each hunted in September, November, and December, and in four animals hunted in January. The unconditional exact test showed that the detection of *S. rileyi* in September–January (15.5%, 95% CI = 8.5%–25.9%) was significantly higher (*p* = 0.0034) than in the February–April period (0%, 95% CI = 0%–8.5%). Meanwhile, *Sarcocystis* sp. LT-2022 was established in two animals collected in March and April. 

### 3.3. Phylogenetic Relationships of Identified Sarcocystis Species

**Figure 3:**
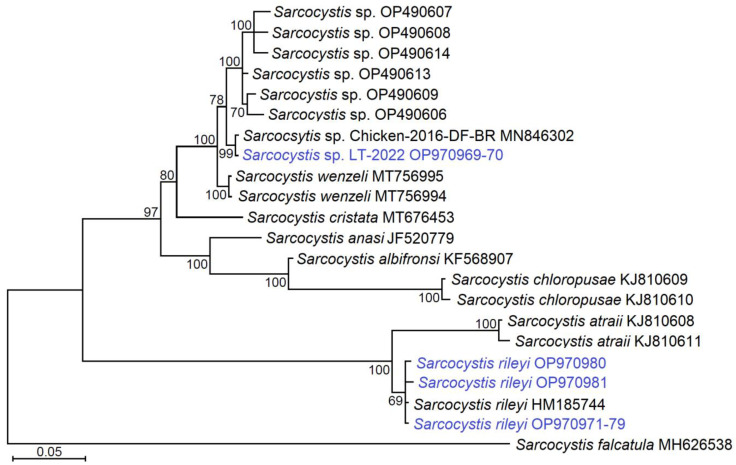
The phylogenetic tree of the *Sarcocystis* species based on *ITS1* sequences and Bayesian inference. The multiple-sequence alignment contained 609 aligned nucleotide positions; the Kimura’s two-parameter substitution model (K2P) [37] was used for analysis. The tree was rooted on *S*. *falcatula* and scaled according to branch length. The posterior probability values supporting branching are shown next to the branches. The sequences determined in the present study are presented in blue.

Comparing the *ITS1* fragments established in the current work, sequences of *Sarcocystis* sp. were longer at the 5’ end and the sequences of *S*. *rileyi* were longer at the 3’ end. Thus, after multiple alignment and sequence truncation, 547 bp-long sequences of *S*. *rileyi* and 554 bp-long sequences of *Sarcocystis* sp. were used for phylogenetic analysis. In the phylogenetic tree, *S*. *rileyi* obtained from mustelids grouped with other *S*. *rileyi* isolates obtained from various intermediate hosts (Figure 3). Based on *ITS1*, *S*. *rileyi* was the sister taxon to *S*. *atraii* and these two species formed separate clusters in the phylogram. 

Based on the analysed *ITS* fragment, *Sarcocystis* sp. LT-2022 isolated from two representatives of two mustelid species were identical to *Sarcocystis* sp. Chicken-2016-DF-BR isolated from chickens in Brazil and they were placed in one well-supported cluster together with *S*. *wenzeli* and *Sarcocystis* sp. from chickens from Malaysia. Whereas, *S*. *cristata* described in the muscles of the great blue turaco (*Corythaeola cristata*) was the sister taxon to the *Sarcocystis* isolates established in Brazil, Malaysia, and Lithuania, and *S*. *wenzeli*. The remaining *Sarcocystis* spp., *S*. *albifornsi*, *S*. *anasi* from anseriforms, and *S*. *chloropusae* from the common moorhen (*Gallinula chloropus*) made a separate cluster in the phylogenetic tree. 

## 4. Discussion

### 4.1. The Role of Mustelids in Distribution of Sarcocystis Species

In the present study, free sporocysts, sporulating oocysts, and loose oocysts were found in the intestinal mucosa of the five examined species, American mink, European pine marten, European polecat, European badger, and beech marten (Figure 1 and Table 1). The morphometric sizes of parasite stages detected in five hosts overlapped. Thus, it was impossible to determine whether the studied host species were infected with the same or different *Sarcocystis* species. Furthermore, it is known that predators can be simultaneously infected with sporocysts of several *Sarcocystis* species [16,24,38]. Therefore, the identification of *Sarcocystis* species was performed using molecular methods. 

Based on the nested PCR and subsequent BLAST analyses of the obtained DNA sequences, two *Sarcocystis* species using birds as intermediate hosts were confirmed. The prevalence of *Sarcocystis* spp. defined by means of molecular examination was relatively low, reaching 11.3% (13/115). By microscopical analysis, sporocysts and/or oocysts of *Sarcocystis* spp. were noticed in more than half (53.0%, 61/115) of the investigated samples. Thus, the obtained results of the present work indicate that the examined mustelids spread considerably more than *Sarcocystis* species, employing mammals rather than birds as their definitive hosts. Our previous research on the small intestine samples of mustelids by species-specific PCR revealed a high prevalence (89.3%, 75/84) of *Sarcocystis* species using cattle as their intermediate hosts [24]. Furthermore, 32 of the 40 (80.0%) examined small intestine samples of American mink tested positive for *S*. *elongata*, *S*. *entzerothi*, *S*. *japonica*, *S*. *silva*, and *S*. *truncata* by molecular methods, producing sarcocysts in muscles of ungulates of the family Cervidae [16]. It was also confirmed by experimental infection that possible definitive hosts of *S*. *campestris*, *S. citellivulpes*, *S. muris*, *S. putorii*, and *S. undulati* are members of family Mustelidae [39]. Research carried out until now implies that mustelids play a significant role for the transmission of various *Sarcocystis* species using hosts that belong to different taxonomic groups.

### 4.2. Mustelids as Possible Definitive Hosts of S. rileyi 

Eleven *ITS1* sequences obtained in the current study demonstrated 99.18%–100% similarity to the sequences of *S*. *rileyi* available in GenBank and showed less than 93% similarity with any other known species of *Sarcocystis*. Hence, *S*. *rileyi* was confirmed in the intestinal mucosa of eight American minks, two European polecats and single European badger (Table 2). The overall prevalence of *S*. *rileyi* in the analysed samples of mustelids was 9.6% (95% CI = 5.1%–16.4%). In the present study, the identified *S*. *rileyi* is a well-known *Sarcocystis* species forming macroscopic sarcocysts resembling grains of rice in the muscles of ducks. This species was described in the late nineteenth century [40] and redescribed in 2003, providing detailed morphological characterisation [41]. For a long time, macrocysts in numerous duck species were recorded only in North America [42,43,44,45,46,47,48]. Based on light microscopy, transmission electron microscopy, and molecular characterisation at three genetic loci (*18S* rDNA, *28S* rDNA, and *ITS1*), *S*. *rileyi* was identified in Lithuania in 2011 [12]. According to the current data, the distribution of *S*. *rileyi* covers the eastern, northern, and central parts of Europe [12,13,29,49,50,51,52,53]. In this continent, *S*. *rileyi* was mostly recorded in mallard [12,13,49,52] and with much less frequency in several other duck species [29,53]. The mallard is the most abundant species of ducks and also one of most important bird game species in Europe [54,55,56]. *Sarcocystis rileyi* cause economic losses, since hunted duck meat contaminated with macrocysts is not suitable for human consumption [13]. Additionally, severe infection of *S*. *rileyi* may result in weakness of hosts, reduced flying capacity, and infected birds may be more easily caught by predators [57]. The stripped skunk (*Mephitis mephitis*) of the family Mephistidae is an experimentally proved definitive hosts of *S*. *rileyi* in North America [58,59]. This small predatory animal lives only in captivity in Europe [60]. Therefore, for a long time it was unclear which predators are responsible for the spread of *S*. *rileyi* in Europe. Based on molecular analysis, red foxes (*Vulpes vulpes*) and raccoon dogs (*Nyctereutes procyonoides*) of the family Canidae were identified as definitive hosts of *S*. *rileyi* in Lithuania and Germany [38,50]. Hence, based on the findings of previous and current investigations, *S*. *rileyi* is transmitted in Europe by predators of family Canidae and Mustelidae. It should be noted that Mephistidae and Mustelidae families are closely related and together with Ailuridae and Procyonidae compose a superfamily, Musteloidea [61]. Thus, the current findings indicate the co-evolution of *S*. *rileyi* with their definitive hosts. A co-evolution of *Sarcocystis* spp. with their definitive host rather than the intermediate host has been shown in the phylogenetic investigations of various groups of *Sarcocystis* species [15,62,63]. The raccoon (*Procyon lotor*) of the family Procyonidae which is native to North America is now spreading in Lithuania through the western part of the country [64]. Taking into account the close relationship of raccoon with mustelids and mephistids, this invasive predator should be screened for the distribution of *S*. *rileyi*.

In general, *Sarcocystis* species are more host-specific for their intermediate hosts than for their definitive hosts. For instance, *S*. *cruzi*, the most common species of *Sarcocystis* of cattle worldwide, is transmissible via dogs, coyotes, foxes, and wolves [1]. With the exception of *S*. *wenzeli*, *Sarcocystis* species transmitted by canids cannot be transmitted by felids and vice versa [65]. However, laboratory experiments evidenced that some *Sarcocystis* spp. transmitted via canids or felids can be spread via mustelids [39,66]. Furthermore, on the basis of molecular investigations of small intestine samples it was shown that *S*. *cruzi* can be spread not only by canids, but also by mustelids [24]. The current study on the basis of *ITS1* sequence analysis also indicates that *S*. *rileyi* can be transmitted in Europe by the members of two families, Canidae and Mustelidae. 

It should be emphasised that in the present study *S*. *rileyi* was identified in mustelids, which were hunted from September to January (15.5%, 11/71). This *Sarcocystis* species was mostly confirmed in American mink, while *S*. *rileyi* was not detected in 44 animals which were collected during February–April (Figure 2). In Lithuania, during September, large flocks of ducks and waders concentrate in partly drained fishponds and these birds form a large part of the diet of invasive American mink [67]. In summer–early autumn, birds are also an important food component for other mustelid species in various regions of Lithuania [18]. Whereas, in late autumn and winter mustelids only occasionally hunt ducks [18,19,20,28,68,69,70]. Oocysts/sporocysts of *Sarcocystis* spp. are found in the faeces of definitive hosts 7–14 days post infection and excretion of infective parasite stages mostly lasts several months [1]. Thus, the observed variations in the identification of *S*. *rileyi* during different months are congruent with the diet of mustelids and the life cycle peculiarities of *Sarcocystis* parasites. The results of the abundance of *S*. *rileyi* depending on the season mustelids were hunted are in congruent with the investigation of *S*. *rileyi* in canids. During a previous study conducted in Lithuania, *S*. *rileyi* was identified in the small intestines of red foxes and raccoon dogs hunted in November and December, but the parasite was not detected in animals hunted in February and March [50]. Thus, future research on the prevalence of *Sarcocystis* in predatory mammals through different seasons is needed. 

### 4.3. Detection of Sarcocystis sp. Closely Related to S. wenzeli in Small Intestine of Mustelids

Based on the obtained *ITS1* sequence analysis, *Sarcocystis* sp. was identified in a single American mink and in a single American pine marten (Table 2). Two 817 bp-long *ITS1* sequences were 100% identical to the sequence of *Sarcocystis* sp. Chicken-2016-DF-BR obtained from the brains of two chickens in the midwest of Brazil [36]. This parasite caused fatal outcomes in backyard chickens. The infected chickens suffered from anorexia, weight loss, incoordination, ataxia, and opisthotonos. The histopathological analysis showed necrotizing granulomatous and meningoencephalitis with intralesional *Sarcocystis*-like schizonts and merozoites. Infected chickens remained free during the day and were kept in the coop at night [36]. It should be noted that severe myositis and encephalitis associated with *Sarcocystis* parasites has been reported several times in domestic fowl in different geographical regions [71,72].

The *ITS1* region is highly variable for *Sarcocystis* spp. [8]. Due to the large number of indels (insertions/deletions) it is hard to align *ITS1* sequences of *Sarcocystis* spp. sharing relatively low similarity. Therefore, the *ITS1* region is not a good choice for the discriminating phylogenetic relationships of genetically remote group of *Sarcocystis*. However, this genetic locus is suitable for phylogenetic analysis of closely related *Sarcocystis* species [63,73]. Several studies have demonstrated that *ITS1* is an appropriate genetic marker inferring phylogenetic relationships of *Sarcocystis* spp. using birds as intermediate hosts [5,9,10,11,73,74,75,76,77,78]. Furthermore, for this group of *Sarcocystis* species, *ITS1* and *28S* rDNA give congruous topology [73,79]. Based on the *ITS1* sequence analysis conducted in the current study, the topology of the examined *Sarcocystis* species using birds–predatory mammals in their intermediate–definitive host life cycle (Figure 3) in general corresponded to that determined in the latest phylogenetic studies [5,36]. *Sarcocystis* sp. LT-2022 obtained in the present work and *Sarcocystis* sp. Chicken-2016-DF-BR were placed in one phylogenetic cluster together with *S*. *wenzeli* infecting chickens and *Sarcocystis* sp. isolated from pooled various tissue samples of native village chickens in Malaysia (Figure 3). Thus, the obtained sequences were grouped with those of *Sarcocystis* spp. parasitising chickens. Further molecular studies are needed to clarify the number of species that represent *S*. *wenzeli*, *Sarcocystis* sp. Chicken-2016-DF-BR, *Sarcocystis* sp. from Malaysian chickens, and *Sarcocystis* sp. LT-2022 identified in the present work. 

There is ongoing debate on the classification of *Sarcocystis* species in chickens [39]. In the latest taxonomic review of the genus *Sarcocystis*, two species infecting chickens, *S*. *horvathi* and *S*. *wenzeli*, were distinguished [1]. *Sarcocystis wenzeli* is characterised morphologically in detail and based on transmission experiments dogs and cats are confirmed as definitive hosts of this species [65], whereas the definitive hosts of *S. horvathi* are unknown [1].

The data of the current work indicate that mustelids might be involved in the transmission of *Sarcocystis* species infecting domestic gallinaceous fowl. However, laboratory infection experiments are definitely necessary to test the results obtained. Additionally, here we present the first identification of *Sarcocystis* sp. in Lithuania, closely related to *Sarcocystis* parasitizing chickens. Despite extensive studies of *Sarcocystis* conducted in Lithuania in various groups of wild birds [7,8,12,73,75,76,77,78,79], these parasites have not been studied in poultry so far. In Lithuania, chickens are mainly raised in poultry farms [80]. However, in rural regions small numbers of domestic fowl are kept free. The main predators of backyard chickens in Lithuania are mustelids and red fox [18]. Mustelids can potentially cause the transmission of highly pathogenic *Sarcocystis* species in poultry farming.

## 5. Conclusions

Based on the nested PCR and sequencing of the *ITS1* region, *S*. *rileyi* producing macroscopic sarcocysts in muscles of ducks was for the first time confirmed in small intestine scrapings of three mustelid species collected in Lithuania. The prevalence of *S*. *rileyi* in the examined mustelids was 9.6%. According to the current data obtained by molecular investigations, canids and mustelids are responsible for the spread of *S*. *rileyi* in Europe. 

Undescribed *Sarcocystis* sp. LT-2022 showed 100% similarity within the 817 bp-long *ITS1* fragment with *Sarcocystis* sp. Chicken-2016-DF-BR, which caused a fatal infection in two backyard chickens in Brazil. The detected *Sarcocystis* parasite was most closely related to *S*. *wenzeli* and *Sarcocystis* sp. using chickens as their intermediate hosts. Thus, this is the first report of *Sarcocystis* sp. associated with possible infection in gallinaceous birds in Lithuania. 

## Figures and Tables

**Table 1:** Detection rates and morphology of oocysts/sporocysts found in small intestine mucosal scrapings of Mustelidae species from Lithuania.

Host Species	Microscopical Detection of *Sarcocystis* spp.		The Size of Sporocysts	The Size of Sporulating Oocysts	The Size of Free Oocysts
	Infected/Investigate (%)	95% CI			
American mink	31/61 (50.8%)	38.5–63.2	10.2–14.1 × 7.1–9.4 (12.3 × 8.3; *n* = 170)	14.5–21.1 × 10.9–17.5 (18.0 × 14.0; *n* = 30)	14.8–23.5 × 13.5–22.4 (21.1 × 18.3; *n* = 14)
European pine marten	15/26 (57.7%)	38.3–75.4	10.1–14.1 × 6.9–10.1 (11.6 × 8.2; *n* = 115)	12.4–19.2 × 10.1–18.3 (14.9 × 12.8; *n* = 20)	17.9–23.1 × 15.5–21.5 (21.8 × 17.6; *n* = 17)
European polecat	11/18 (61.1%)	37.4–81.5	10.0–14.6 × 6.7–9.9 (12.4 × 8.3; *n* = 140)	13.3–19.5 × 11.1–18.0 (17.5 × 13.3; *n* = 40)	13.9–23.0 × 12.0–22.0 (19.2 × 18.5; *n* = 15)
European badger	1/6 (16.7%)	8.6–58.9	10.0–14.1 × 6.4–9.6 (12.7 × 8.1; *n* = 15)	13.5–18.6 × 9.7–16.0 (16.2 × 13.1; *n* = 17)	-
Beech marten	3/4 (75.0%)	24.9–98.7	7.0–12.6 × 7.0–8.6 (10.2 × 7.8; *n* = 10)	13.5–23.9 × 10.1–17.5 (16.2 × 13.1; *n* = 23)	-
Overall	61/115 (53.0%)	43.9–62.2	7.0–14.6 × 6.4–10.2 (12.1 × 8.2; *n* = 450)	12.4–23.9 × 9.7–18.3 (16.5 × 13.2; *n* = 130)	13.9–23.5 × 12.0–22.2 (20.6 × 17.4; *n* = 46)

**Table 2:** Molecular identification of two avian *Sarcocystis* species in the mucosal scrapings of the examined mustelids.

Host Species	N	*Sarcocystis rileyi* (%, 95% CI)	*Sarcocystis* sp. (%, 95% CI)
American mink	61	8 (13.1, 6.2–24.4)	1 (1.6, 0.9–8.7)
European pine marten	26	0	1 (3.8, 0.2–18.8)
European polecat	18	2 (11.1, 20.0–33.0)	0
European badger	6	1 (16.7, 8.6–58.9)	0
Beech marten	4	0	0
Overall	115	11 (9.6, 5.1–16.4)	2 (3.3, 0.3–6.3)

## Data Availability

The *ITS1* sequences of *S*. *rileyi* and *Sarcocystis* sp. were submitted in GenBank database with accession numbers OP970969–OP970981.

## References

[1] Dubey J.P., Calero-Bernal R., Rosenthal B.M., Speer C.A., Fayer R. (2015). Sarcocystosis of Animals and Humans.

[2] Mehlhorn H., Heydorn A.O. (1978). The Sarcosporidia (Protozoa, Sporozoa): Life Cycle and Fine Structure. Adv. Parasitol..

[3] Tenter A.M. (1995). Current research on *Sarcocystis* species of domestic animals. Int. J. Parasitol..

[4] Juozaitytė-Ngugu E., Švažas S., Šneideris D., Rudaitytė-Lukošienė E., Butkauskas D., Prakas P. (2021). The Role of Birds of the Family Corvidae in Transmitting *Sarcocystis* Protozoan Parasites. Animals.

[5] Máca O., González-Solís D. (2021). *Sarcocystis cristata* sp. nov. (Apicomplexa, Sarcocystidae) in the imported great blue turaco *Corythaeola cristata* (Aves, Musophagidae). Parasites Vectors.

[6] Gallo S.S.M., Lindsay D.S., Ederli N.B., Matteoli F.P., Venancio T.M., de Oliveira F.C.R. (2018). Identification of opossums *Didelphis aurita* (Wied-Neuweid, 1826) as a definitive host of *Sarcocystis falcatula*-like sporocysts. Parasitol. Res..

[7] Kutkienė L., Prakas P., Sruoga A., Butkauskas D. (2012). Description of *Sarcocystis anasi* sp. nov. and *Sarcocystis albifronsi* sp. nov. in birds of the order Anseriformes. Parasitol. Res..

[8] Prakas P., Oksanen A., Butkauskas D., Sruoga A., Kutkienė L., Švažas S., Isomursu M., Liaugaudaitė S. (2014). Identification and Intraspecific Genetic Diversity of *Sarcocystis rileyi* from Ducks, *Anas* spp., in Lithuania and Finland. J. Parasitol..

[9] El-Morsey A., El-Seify M., Desouky A.-R.Y., Abdel-Aziz M.M., El-Dakhly K.M., Kasem S., Abdo W., Haridy M., Sakai H., Yanai T. (2015). Morphologic and molecular characteristics of *Sarcocystis atraii* n. sp. (Apicomplexa: Sarcocystidae) infecting the common coot (*Fulica atra*) from Egypt. Acta Parasitol..

[10] El-Morsey A., El-Seify M., Desouky A.Y., Abdel-Aziz M.M., Sakai H., Yanai T. (2015). *Sarcocystis chloropusae* (protozoa: Sarcocystidae) n. sp. from the common moorhen (*Gallinula chloropus*) from Egypt. Parasitology.

[11] Pan J., Ma C., Huang Z., Ye Y., Zeng H., Deng S., Hu J., Tao J. (2020). Morphological and molecular characterization of *Sarcocystis wenzeli* in chickens (*Gallus gallus*) in China. Parasites Vectors.

[12] Kutkienė L., Prakas P., Sruoga A., Butkauskas D. (2011). Identification of *Sarcocystis rileyi* from the mallard duck (*Anas platyrhynchos*) in Europe: Cyst morphology and results of DNA analysis. Parasitol. Res..

[13] Zuo S., Sørensen S.R., Kania P.W., Buchmann K. (2021). *Sarcocystis rileyi* (Apicomplexa) in *Anas platyrhynchos* in Europe with a potential for spread. Int. J. Parasitol. Parasites Wildl..

[14] Prakas P., Kirillova V., Gavarāne I., Grāvele E., Butkauskas D., Rudaitytė-Lukošienė E., Kirjušina M. (2019). Morphological and molecular description of *Sarcocystis ratti* n. sp. from the black rat (*Rattus rattus*) in Latvia. Parasitol. Res..

[15] Gjerde B. (2013). Phylogenetic relationships among *Sarcocystis* species in cervids, cattle and sheep inferred from the mitochondrial cytochrome c oxidase subunit I gene. Int. J. Parasitol..

[16] Prakas P., Rudaitytė-Lukošienė E., Šneideris D., Butkauskas D. (2021). Invasive American mink (*Neovison vison*) as potential definitive host of *Sarcocystis elongata*, *S. entzerothi*, *S. japonica*, *S. truncata* and *S. silva* using different cervid species as intermediate hosts. Parasitol. Res..

[17] Prakas P., Butkauskas D., Švažas S., Stanevičius V. (2018). Morphological and genetic characterisation of *Sarcocystis halieti* from the great cormorant (*Phalacrocorax carbo*). Parasitol. Res..

[18] Kontrimavičius V. (1988). Lietuvos Fauna: Žinduoliai.

[19] Balčiauskas L., Trakimas G., Juškaitis R., Ulevičius A., Balčiauskienė L. (1999). Atlas of Lithuanian Mammals, Amphibians and Reptiles.

[20] Baghli A., Engel E., Verhagen R. (2002). Feeding habits and trophic niche overlap of two sympatric mustelidae, the polecat *Mustela putorius* and the beech marten *Martes foina*. Z. Für Jagdwiss..

[21] Koepfli K.-P., Deere K.A., Slater G.J., Begg C., Begg K., Grassman L., Lucherini M., Veron G., Wayne R.K. (2008). Multigene phylogeny of the Mustelidae: Resolving relationships, tempo and biogeographic history of a mammalian adaptive radiation. BMC Biol..

[22] Newman C., Zhou Y.-B., Buesching C.D., Kaneko Y., Macdonald D.W. (2011). Contrasting Sociality in Two Widespread, Generalist, Mustelid Genera, *Meles* and *Martes*. Mammal Study.

[23] Law C.J., Slater G.J., Mehta R.S. (2018). Lineage Diversity and Size Disparity in Musteloidea: Testing Patterns of Adaptive Radiation Using Molecular and Fossil-Based Methods. Syst. Biol..

[24] Prakas P., Balčiauskas L., Juozaitytė-Ngugu E., Butkauskas D. (2021). The Role of Mustelids in the Transmission of *Sarcocystis* spp. Using Cattle as Intermediate Hosts. Animals.

[25] Bonesi L., Palazon S. (2007). The American mink in Europe: Status, impacts, and control. Biol. Conserv..

[26] Holopainen S., Väänänen V.-M., Vehkaoja M., Fox A.D. (2021). Do alien predators pose a particular risk to duck nests in Northern Europe? Results from an artificial nest experiment. Biol. Invasions.

[27] Viksne J., Švažas S., Czajkowski A. (2010). Atlas of Duck Populations in Eastern Europe.

[28] Baltrūnaitė L. (2002). Diet Composition of the Red Fox (*Vulpes Vulpes* L.), Pine Marten (*Martes Martes* L.) and Raccoon Dog (*Nyctereutes Procyonoides* Gray) in Clay Plain Landscape, Lithuania. Acta Zool. Litu..

[29] Gjerde B. (2014). Molecular characterisation of *Sarcocystis rileyi* from a common eider (*Somateria mollissima*) in Norway. Parasitol. Res..

[30] Rozen S., Skaletsky H. (2000). Primer3 on the WWW for general users and for biologist programmers. Bioinform. Methods Protoc..

[31] Kumar S., Stecher G., Tamura K. (2016). MEGA7: Molecular Evolutionary Genetics Analysis Version 7.0 for Bigger Datasets. Mol. Biol. Evol..

[32] Milne I., Wright F., Rowe G., Marshall D., Husmeier D., McGuire G. (2004). TOPALi: Software for automatic identification of recombinant sequences within DNA multiple alignments. Bioinformatics.

[33] Reiczigel J. (2003). Confidence intervals for the binomial parameter: Some new considerations. Stat. Med..

[34] Reiczigel J., Abonyi-Tóth Z., Singer J. (2008). An exact confidence set for two binomial proportions and exact unconditional confidence intervals for the difference and ratio of proportions. Comput. Stat. Data Anal..

[35] Rózsa L., Reiczigel J., Majoros G. (2000). Quantifying Parasites in Samples of Hosts. J. Parasitol..

[36] Wilson T.M., Sousa S.K., Paludo G.R., de Melo C.B., Llano H.A., Soares R.M., Castro M.B. (2020). An undescribed species of *Sarcocystis* associated with necrotizing meningoencephalitis in naturally infected backyard chickens in the Midwest of Brazil. Parasitol. Int..

[37] Kimura M. (1980). A simple method for estimating evolutionary rates of base substitutions through comparative studies of nucleotide sequences. J. Mol. Evol..

[38] Moré G., Maksimov A., Conraths F., Schares G. (2016). Molecular identification of *Sarcocystis* spp. in foxes (*Vulpes vulpes*) and raccoon dogs (*Nyctereutes procyonoides*) from Germany. Veter Parasitol..

[39] Odening K. (1998). The present state of species-systematics in *Sarcocystis Lankester*, 1882 (Protista, Sporozoa, Coccidia). Syst. Parasitol..

[40] Stiles C.W. (1893). On the presence of sarcosporidia in birds. USDA Bur. Anim. Ind. Bull..

[41] Dubey J.P., Cawthorn R.J., Speer C.A., Wobeser G.A. (2003). Redescription of the sarcocysts of *Sarcocystis rileyi* (Apicomplexa: Sarcocystidae). J. Eukaryot. Microbiol..

[42] Erickson A.B. (1940). *Sarcocystis* in Birds. Auk.

[43] Cornwell G. (1963). New Waterfowl Host Records for *Sarcocystis rileyi* and a Review of Sarcosporidiosis in Birds. Avian Dis..

[44] Chabreck R.H. (1965). Sarcosporidiosis in Ducks in Louisiana. Trans. N. Am. Wildl. Conf..

[45] Drouin T.E., Mahrt J.L. (1979). The Prevalence of *Sarcocystis* Lankester, 1882, in some Bird Species in Western Canada, with Notes on its Life Cycle. Can. J. Zool..

[46] Fedynich A.M., Pence D.B. (1992). *Sarcocystis* in Mallards on the Southern High Plains of Texas. Avian Dis..

[47] Dubey J.P., Rosenthal B.M., Felix T.A. (2010). Morphologic and Molecular Characterization of the Sarcocysts of *Sarcocystis rileyi* (Apicomplexa: Sarcocystidae) from the Mallard Duck (*Anas platyrhynchos*). J. Parasitol..

[48] Padilla-Aguilar P., Romero-Callejas E., Osorio-Sarabia D., Ramírez-Lezama J., Cigarroa-Toledo N., Machain-Williams C., Manterola C., Zarza H. (2016). Detection and Molecular Identification of *Sarcocystis rileyi* (Apicomplexa: Sarcocystidae) from a Northern Shoveler (*Anas clypeata*) in Mexico. J. Wildl. Dis..

[49] Kalisinska E., Betlejewska K.M., Schmidt M., Gozdzicka-Jozefiak A., Tomczyk G. (2003). Protozoal Macrocysts in the Skeletal Muscle of a Mallard duck in Poland: The First Recorded Case. Acta Parasitol..

[50] Prakas P., Liaugaudaitė S., Kutkienė L., Sruoga A., Švažas S. (2015). Molecular identification of *Sarcocystis rileyi* sporocysts in red foxes (*Vulpes vulpes*) and raccoon dogs (*Nyctereutes procyonoides*) in Lithuania. Parasitol. Res..

[51] Cromie R., Ellis M. (2019). *Sarcocystis* Survey. *Sarcocystis* Survey Feedback Report–The UK Wildfowl *Sarcocystis* Survey. www.sarcocystissurvey.org.uk/2015-2018-feedback-report/.

[52] Szekeres S., Juhász A., Kondor M., Takács N., Sugár L., Hornok S. (2019). *Sarcocystis rileyi* emerging in Hungary: Is rice breast disease underreported in the region?. Acta Vet. Hung..

[53] Muir A., Ellis M., Blake D.P., Chantrey J., Strong E.A., Reeves J.P., Cromie R.L. (2020). *Sarcocystis rileyi* in UK free-living wildfowl (Anatidae): Surveillance, histopathology and first molecular characterisation. Vet. Rec..

[54] Mooij J.H. (2005). Protection and use of Waterbirds in the European Union. Beitr. Jagd Wildforschung.

[55] Hirschfeld A., Attard G., Scott L. (2019). Bird Hunting in Europe: An Analysis of Bag Figures and the Potential Impact on the Conservation of Threatened Species. Br. Birds.

[56] Sibille S., Griffin C., Scallan D. (2020). Europe’s Huntable Birds: A Review of Status and Conservation Priorities. European Federation for Hunting and Conservation (FACE). https://www.face.eu/.

[57] Friend M., Franson J.C. (1999). Field Manual of Wildlife Diseases—General Field Procedures and Diseases of Birds.

[58] Cawthorn R.J., Rainnie D., Wobeser G. (1981). Experimental transmission of *Sarcocystis* sp. (Protozoa: Sarcocystidae) between the shoveler (*Anas clypeata*) duck and the striped skunk (*Mephitis mephitis*). J. Wildl. Dis..

[59] Wicht R.J. (1981). Transmission of *Sarcocystis rileyi* to the striped skunk (*Mephitis mephitis*). J. Wildl. Dis..

[60] Wilson D.E., Reeder D.M. (2005). Mammal Species of the World: A Taxonomic and Geographic Reference.

[61] Flynn J.J., Finarelli J.A., Zehr S., Hsu J., Nedbal M.A. (2005). Molecular Phylogeny of the Carnivora (Mammalia): Assessing the Impact of Increased Sampling on Resolving Enigmatic Relationships. Syst. Biol..

[62] Šlapeta J.R., Modrý D., Votýpka J., Jirků M., Lukeš J., Koudela B. (2003). Evolutionary relationships among cyst-forming coccidia Sarcocystis spp. (Alveolata: Apicomplexa: Coccidea) in endemic African tree vipers and perspective for evolution of heteroxenous life cycle. Mol. Phylogenet. Evol..

[63] Morrison D.A., Bornstein S., Thebo P., Wernery U., Kinne J., Mattsson J.G. (2004). The current status of the small subunit rRNA phylogeny of the coccidia (Sporozoa). Int. J. Parasitol..

[64] Jasiulionis M., Stirkė V., Balčiauskas L. (2022). Invasive Raccoon Dog (*Nyctereutes procyonoides*) and Raccoon (*Procyon lotor*) Monitoring in Lithuania Based on Camera Traps Data. Biol. Life Sci. Forum.

[65] Mao J.B., Zuo Y.X. (1994). Studies on the Prevalence and Experimental Transmission of *Sarcocystis* sp. in Chickens. Acta Vet. Zootech Sin..

[66] Pak S.M., Perminova V.V., Yeshtokina N.V., Beyer T.V., Bezukladnikova N.A., Galuzo I.G., Konovalova S.I., Pak S.M. (1979). Sarcocystis citellivulpes sp. n. from the Yellow Suslik *Citellus fulvus* Lichtenstain, 1923. Toksoplazmidy, Protozoologiya.

[67] Švažas S., Kozulin A. (2002). Waterbirds of Large Fishponds of Belarus and Lithuania.

[68] Lanszki J., Heltai M. (2011). Feeding Habits of Sympatric Mustelids in an Agricultural Area of Hungary. Acta Zool. Acad. Sci. Hung..

[69] Zschille J., Stier N., Roth M., Mayer R. (2013). Feeding habits of invasive American mink (*Neovison vison*) in northern Germany—Potential implications for fishery and waterfowl. Acta Theriol..

[70] Tsunoda H., Peeva S., Raichev E., Kronawetter T., Kirilov K.B., Georgiev D., Kaneko Y. (2022). Patterns of spatial distribution and diel activity in carnivore guilds (Carnivora). J. Vertebr. Biol..

[71] Munday B.L., Humphrey J.D., Kila V. (1977). Pathology Produced by, Prevalence, of, and Probable Life-cycle of a Species of *Sarcocystis* in the Domestic Fowl. Avian Dis..

[72] Mutalib A., Keirs R., Maslin W., Topper M., Dubey J.P. (1995). *Sarcocystis*-Associated Encephalitis in Chickens. Avian Dis..

[73] Prakas P., Kutkienė L., Butkauskas D., Sruoga A., Žalakevičius M. (2013). Molecular and morphological investigations of *Sarcocystis corvusi* sp. nov. from the jackdaw (*Corvus monedula*). Parasitol. Res..

[74] Olias P., Olias L., Lierz M., Mehlhorn H., Gruber A.D. (2010). *Sarcocystis calchasi* is distinct to *Sarcocystis columbae* sp. nov. from the wood pigeon (*Columba palumbus*) and *Sarcocystis* sp. from the sparrowhawk (*Accipiter nisus*). Vet. Parasitol..

[75] Kutkienė L., Prakas P., Butkauskas D., Sruoga A. (2012). Description of *Sarcocystis turdusi* sp. nov. from the common blackbird (*Turdus merula*). Parasitology.

[76] Prakas P., Butkauskas D., Švažas S., Juozaitytė-Ngugu E., Stanevičius V. (2018). Morphologic and genetic identification of *Sarcocystis fulicae* n. sp.(Apicomplexa: Sarcocystidae) from the Eurasian coot (*Fulica atra*). J. Wildl. Dis..

[77] Prakas P., Butkauskas D., Juozaitytė-Ngugu E. (2020). Molecular identification of four *Sarcocystis* species in the herring gull, *Larus argentatus*, from Lithuania. Parasites Vectors.

[78] Juozaitytė-Ngugu E., Butkauskas D., Švažas S., Prakas P. (2022). Investigations on *Sarcocystis* species in the leg muscles of the bird family Corvidae in Lithuania. Parasitol. Res..

[79] Prakas P., Butkauskas D., Juozaitytė-Ngugu E. (2020). Molecular and morphological description of *Sarcocystis kutkienae* sp. nov. from the common raven (*Corvus corax*). Parasitol. Res..

[80] Ministry of Agriculture of the Republic of Lithuania (2020). Lithuanian Agrifood Sector. https://zum.lrv.lt/uploads/zum/documents/files/LT_versija/Naujiena/Leidiniai/Lithuanian_agrifood_sector_2020.pdf.

